# How scientists interpret and address funding criteria: value creation and undesirable side effects

**DOI:** 10.1007/s11187-022-00697-4

**Published:** 2022-10-10

**Authors:** Conor O’Kane, Jing A. Zhang, Jarrod Haar, James A. Cunningham

**Affiliations:** 1 Otago Business School, University of Otago, Dunedin, New Zealand; 2 Auckland University of Technology Business School, Auckland University of Technology, Auckland, New Zealand; 3 Newcastle University Business School, Newcastle University, Newcastle Upon Tyne, UK; 4 Centre for Innovation Research, Lund University, Lund, Sweden

**Keywords:** Science funding, Signal theory, Resourcefulness, Value creation, Principal investigators, O30, O31, O38

## Abstract

Scientists and funding bodies are interdependent actors involved in an ongoing two-way signalling interaction; however, we lack insight on the social mechanisms underpinning this interaction. To address this issue, we examine how successfully funded scientists interpret and address criteria set by the funding body to maximise their chances of funding success. We also consider the possible adverse side effects that can arise from scientists’ competitive efforts to address these criteria. Our findings identify a portfolio of funding criteria—research feasibility, research alignment and team credentials—that scientists address when preparing grant applications. Effectively addressing these criteria enhances the prospects of funding success and value creation. However, we also find that scientists can over-address funding criteria, which is counterproductive and yields undesirable side effects. Our research therefore makes an important distinction between the possibilities for value creation and the value creation frictions that can unintentionally arise based on how grant-submitting scientists interpret and address the criteria signalled by the funding body. Our research has implications for policymakers, funding bodies and scientists which we also discuss.

## Abstract (plain language summary)

Hyper-competition for public grant funding can compromise the value created from successfully funded research. When a scientist successfully acquires a publicly funded grant, it sends a signal of research quality and value. However, competition for funding is getting increasingly difficult. Scientists must balance their desire to do the research they want to do against the insistence of funding bodies to have the criteria they set addressed appropriately. Our research shows that, while this two-way interaction between scientists and funding bodies increases the quality and value of funded research, it also stifles research novelty and human capital development. This occurs because scientists over-conform to the perceived funding criteria. The main implication of this research is that policymakers and funding bodies must carefully monitor how they set, communicate and implement funding criteria and evaluation processes. They must ensure there is enough funding dedicated to early-stage and higher risk research, and also account for equality and diversity to support scientists at all career stages.

## Introduction

Underpinning the development of the Pfizer-BioNTech and Moderna COVID-19 vaccines in 2020 was a body of work dating back to the early 1990s on messenger RNA (mRNA) led by Katalin Kariko, a Hungarian biochemist who emigrated to the USA to pursue her science career. An interesting subplot to Kariko’s breakthrough contribution is how often her research progress with mRNA were almost derailed by upheavals in her career and in particular a lack of funding. Kariko’s applications for research grant support were rejected over and over again, failing to successfully acquire a single major grant from the National Institutes of Health throughout her career (Heng, 2021). Consequently, Kariko had to contend with demotions, a lack of career progression and job insecurity as she periodically sought out new labs, supervisors and teams with access to research resources she could not attain herself. According to Kariko, her own work on mRNA work was too unorthodox for the grant funding system “we couldn’t get money then because it was too novel” (Kollewe, 2020). Supporting this view, her close collaborator Dr Drew Weissman commented “we both started writing grants…we didn’t get most of them. People were not interested in mRNA. The people who reviewed the grants said mRNA will not be a good therapeutic, so don’t bother’” (Kolata, 2021). In many respects, the unwavering persistence that underpinned Kariko’s eventual breakthroughs are as impressive as their transformative impact. Nevertheless, in reflecting on Kariko’s achievements and journey, an editorial in the Harvard Crimson argued that science funding mechanisms are failing society, concluding “the fact stands: The system failed to support mRNA research, and most researchers would have pivoted” (Heng, 2021).

While this story has recently received considerable attention, in reality, concerns about what research is (or is not) successful in contestable public grant funding have persisted for a long time. It points to the fundamental, yet poorly understood, issue on how scientists and funding bodies respectively address and set/evaluate funding criteria. In public grant funding, scientists and funding bodies are interdepended actors. The science community not only seeks resources from funding bodies for their research, but they also offer bottom-up input on the shaping of funding calls and in turn respond to these calls once formalised. Funding bodies, on the other hand, rely on the input and expertise of the science community in shaping their funding calls and then evaluate grant proposals submitted by the science community. In effect, scientists and funding bodies are both senders and receivers in an ongoing two-way signalling interaction. However, we lack insight on the social mechanisms underpinning this interaction. More precisely, how funding bodies, through their control and allocation of critical financial resources, influence the cognitive development of science remains unclear (Braun, 1998).

Drawing on signal theory (Connelly et al., 2011; Spence, 1978; Stiglitz, 1985), which details how one actor plausibly conveys information about itself that is otherwise unobservable to another actor, the aim of this research is to examine how scientists put forward optimal attributes in their grant applications that can maximise their chances of funding success. To achieve this research aim, our research has two areas of focus. First, we examine how successfully funded scientists interpret and address the criteria set by the funding body when preparing and submitting their grant proposals. To do this, we also employ the emerging perspective of resourcefulness (Michaelis et al., 2022; Stevenson et al., 2021), which details how individuals and organisations achieve more with less in resource constrained environments. Second, we consider what Scholten et al. (2021) refer to as the “undesired side effects” that can arise in the competition for grant funding. While the value creation potential of the research being proposed is typically quite explicit within grant submissions, less observable and understood are these unwelcome side effects that can deter value creation. Intense competition for funding may force scientists to overly conform to the guidelines set by funding bodies, however, in what way these efforts might detrimentally impact the value created from the funded research has received little attention in the literature. Therefore, we also examine the possible adverse side effects that can arise from scientists’ competitive efforts to address the criteria signalled by the funding body.

Our research utilises an in-depth case study of Health Research Council (HRC) funded scientists in New Zealand (NZ). Our case study draws on extensive secondary data and interviews with over forty scientists that led successfully funded research grants, some of whom also served on funding body review committees. Our findings identify a portfolio of funding criteria—research feasibility, research alignment and team credentials—that scientists address when preparing grant applications. We show how effectively addressing these criteria can enhance the competitiveness of scientists’ grant proposals and this in turn improves prospects of funding success and value creation. However, our research also shows that, given the hyper-competition for funding, scientists can over-address one or more of these criteria which can be counterproductive. The contributions of our research are as follows. First, we show how resourcefulness behaviour at the individual scientist level is fundamental to value creation in public grant funding. Second, our study makes an important distinction between the possibilities for value creation and the value creation frictions that can unintentionally arise in the pursuit of science grant funding. Third, for signal theory, we provide novel insights on the cognitive processes of scientists as signal senders, in particular how they can differentially interpret and address the same (multiple) signal criteria.

The remainder of this paper is structured as follows. In Section 2, we introduce signal theory and its relevance to grant funding and value creation. Section 2.1.3 details the study’s research method and Section 3 presents the research findings. In Section 4, we discuss the contributions of our research for literature and theory. In Section 5, we offer some concluding comments, practical implications and promising avenues for future research.

## Science grant funding as a signal

Signalling theory details how actors acquire and send signals to receivers to overcome information asymmetries and reduce transaction costs (Spence, 1978). A common example would be how college degrees help graduates signal their qualities to employers in the labour market. In line with this example, the theory posits that signals must meet two conditions. First, in order to be trusted and perceived as valuable, signals must be observable (Connelly et al., 2011). Second, signals must be costly or difficult to acquire (Stigliz, 1985). This cost is aligned with otherwise unobservable quality associated with the signal, meaning higher quality actors have an advantage in acquiring the signal (Connelly et al., 2011). In contrast, as lower quality actors will find the criteria associated with acquiring the signal prohibitive or excessive, they are less likely to be successful. In essence, according to signal theory, signals produce a separating equilibrium, enabling signal senders and receivers to distinguish between actors of higher and lower quality (Bergh et al., 2014).

In the context of science funding, competitively acquired research grants awarded by public funding agencies can be considered valuable signals. A separating equilibrium is apparent in how some research proposals are successfully funded and others are not. Research grants are also observable and costly to acquire. Observability is apparent in how grants represent an important “pointing signal” (Bianchi et al., 2019) that provides scientists with a public stamp of approval on the research they are undertaking. Grants positively influence scientists’ visibility and positional power within the field, as well as their ability to do the type of research they want to do and to attract the attention of other prominent research groups or emerging researchers as collaborators (Braun, 1998). As noted by Hicks and Katz (2011, p.149).

Prizes and acclaim alone do not enable a person to conduct further research. Those motivated to continue in research and indeed to compete at the highest level in their fields need the time, equipment and talent that funding buys.

Further supporting the point that successfully funded grants represent observable signals are contributions in the literature that highlight how they positively influence career progression and improve researchers’ status, recognition and collaborative networks (Bloch et al., 2014); reduce search costs for research among peers, industry, media and the general public (Cunningham et al., 2018; O’Kane et al., 2020); help universities to convey institutional prestige and quality (D’Este et al., 2013; Gralka et al., 2019) and allow funding bodies to legitimise their role by showcasing the impactful research they have funded (Lane & Bertuzzi, 2011). Research grants are also difficult or costly for scientists to acquire (Ayoubi et al., 2019; O’Kane et al., 2020). Science funding bodies, similar to the evaluation processes for public R&D subsidies to firms (Bianchi et al., 2019; Chen et al., 2018; Takalo & Tanayama, 2010), incorporate well-designed, resourced and rigorous evaluation procedures involving expert peer-review panels (Braun, 1998). In preparing their grant applications for evaluation, scientists need to compete against peers and address the critical requirements as effectively as they can.

**Fig. 1:**
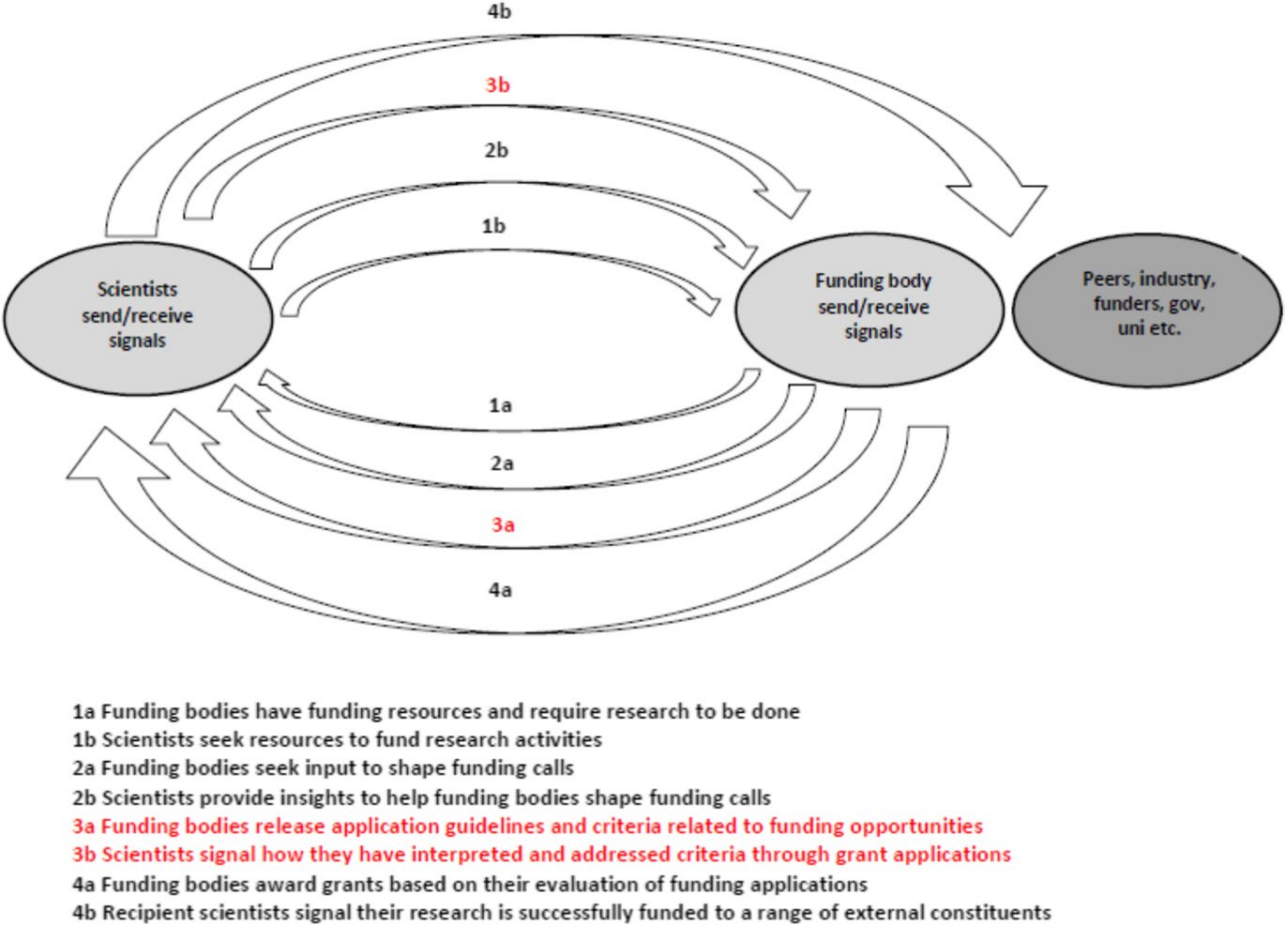
Scientists and funding body two-way signalling process

Building on the idea that research grants in science represent valuable signals, the aim of this research is to examine the process of signal acquisition or the optimal attributes put forward by grant applicants to maximise their chances of funding success. As illustrated in Fig. 1, scientists and funding bodies are both senders and receivers in an ongoing two-way signalling interaction. It is what Steigenberger and Wilhelm (2018) refer to as a “high noise environment” involving multiple signals flowing both ways. On one level, scientists seek resources to undertake their research, while funding bodies require the expertise of the science community to undertake important research activities. On another level, funding bodies look for input and direction in the shaping of funding calls, with the science community being a key source of invaluable insights in this respect. Funding bodies provide some guidelines and application criteria for finalised funding calls and the science community in turn, through their grant applications, signal how they have interpreted and addressed this criteria. Funding bodies award grants based on their evaluation of these applications, with recipient scientists then able to signal to funding bodies (in subsequent funding rounds) and other external constituents that their research is successfully funded. Based on this logic, our research (in red) focuses specifically on how scientists signal how they have interpreted and addressed the criteria set by the funding body. This focus is in line with recent conceptual work (Drover et al., 2018) which argues that greater considerations should be afforded to the cognitive processes of signal actors in the signalling process.

### How scientists address funding criteria: value creation and undesirable side effects

As part of the two-way signalling process between funding bodies and scientists, there is pressure on funding bodies to select suitable projects and programmes from their evaluations. As explained by Braun, (1998, p.811), public funding agencies:

Resemble banks in the economic system: they are investing money into promising projects or are giving credits to trustworthy and (hopefully) innovative scientists. Like banks they have to make sure they are choosing among the right projects and scientists in order to not forego their ‘capital return’…it is a risky enterprise which is based on the hope for future returns by providing ‘capital loans’ to actors in the fields (Braun, 1998, p.811).

An important determinant of the quality of grant applications received by funding bodies are the guidelines and criteria made available. However to date, little if any research has focused in-depth on how the science community interprets and addresses these criteria. This point has support in both the signal and science funding pieces of literature. In the signal literature, there are calls for more research on how signal senders differentially allocate attention to and address signal criteria (Drover et al., 2018). Within the literature on science funding, Scholten et al., (2021, p.2) note that the implications of external science funding on the research practices of scientists remain largely unknown. Drawing on the resourcefulness perspective (Michaelis et al., 2022; Stevenson et al., 2021), we suggest both grant-seeking scientists and the funding providers are likely to pursue resourcefulness. That is they will draw on learned experiences and behaviours that involve “creatively bringing to bear and deploying resources to generate and capture new or unexpected sources of value” (Williams et al., 2021, p. 4). More precisely, it is likely that scientists will look to address funding criteria in creative or differentiated ways that will allow them to demonstrate how they will make effective use (Fisher et al., 2020) and efficiently stretch (Sonenshein, 2017) any resources they acquire. Funders for their part are similarly likely to enact resourcefulness behaviours when evaluating grant proposals to ensure funding is not misdirected or wasted. As it stands however, further research is needed to better understand the practices of scientists pursuing grant funding.

Another gap in the literature pertaining to the acquisition of grant funding relates to the full set of outcomes that are possible from scientists’ efforts to address funding criteria. As argued by Braun (1998), despite being recognised for having a significant influence on the cognitive development of science through the allocation of critical resources, systematic evaluation on the impact of funding bodies is lacking. On the one hand, the value created from the public grant funding process is well recognised. The allocation of funding to higher performing researchers can deepen and sustain their performance and raise the performance of other researchers in the system. There is even evidence that just applying for external grant funding, regardless of whether successful or not, creates value by stimulating greater collaboration and improving the quality and quantity of publication productivity (Ayoubi et al., 2019). On the other hand, less well understood are the “undesired side effects” that can arise in the competition for grant funding (Scholten et al., 2021). While some problems stem from funding body evaluations not being applied in a consistent way (Fang & Casadevall, 2016) or being susceptible to the subjectivity and bounded rationality (Simon, 1972) of reviewers, other detrimental side effects may arise more from the competitive efforts of grant submitting scientists. More precisely, hyper-competition for grant funding can force scientists to over-address or over-conform when responding to the signal criteria and guidelines set by funding bodies; however, this issue in terms of how it might detrimentally impact the value created from grant funding has not being examined in the literature.

Thus, with the aim in this research of examining the optimal attributes put forward by grant applicants to maximise their chances of funding success, our research addresses two specific questions (1) how do scientists signal how they have interpreted and addressed the criteria set by the funding body? and (2) what are some of the adverse side effects that can arise from scientists’ competitive efforts to address this criteria? In the paragraphs that follow, we identify three funding body requirements, or signal criteria, that are emphasised in the literature—(1) feasibility and originality of the research; (2) alignment with funding body’s mission; (3) the credentials of the research team—and consider how scientists’ efforts to effectively address these in their grant application may result in undesirable side effects that hinder value creation.

#### Research feasibility and originality

Fundamentally, scientists’ grant applications are assessed for quality and originality. Key to research originality and innovation is the combination of diverse knowledge based (Nelson & Winter, 1982). In line with this view, scholars report how successful grant applications survey science, market, social and policy developments to envision and articulate new value-creating research trajectories (Cunningham et al., 2015; Kidwell, 2014; Mangematin et al., 2014).

However, research originality must be balanced with evidence of feasibility and validation (Henize et al., 2009). There is ample evidence that, given the high levels of competition for funding (Fang et al., 2016; Whitley et al., 2018), these expectations around feasibility and plausibility can trump research originality, making both grant submitting scientists and evaluating review panels more cautious and risk averse, which can in turn can constrain the development and funding of path-breaking and highly innovative projects (Charlton, 2009; Laudel, 2006; Laudel & Gläser, 2014). Case in point would be the mRNA grant applications by Kariko, which were largely assessed as being too original and risky to fund. It is unsurprising then that O’Kane et al.’s (2015) study on the strategies employed by successfully funded scientists found that three of the four grant writing strategies identified reinforced existing science trajectories. Further supporting this point, Banal-Estañol et al. (2019) find that funding bodies regard grant proposals with unusually diverse knowledge and expertise as less safe. Chai and Menon (2019) argue that while mainstream or familiar research ideas are more likely to lead to successful grants, they are also less likely to be impactful in the value they create. Boudreau et al. (2016) show how highly novel research proposals are scored lower by grant evaluators. Finally, Azoulay et al. (2011) show that funding bodies which reward long-term success and value creation by accepting early failure and encouraging experimentation and flexibility are more likely to produce breakthrough innovations; however, such tolerance within funding bodies is far from the norm.

#### Alignment with funding body mission

In preparing grant applications, scientists must be cognizant that their proposed research conforms and aligns to the mission and programme criteria of the funding body (Braun, 1998; O’Kane et al., 2015). In the case of public funding agencies, evaluations of grant applications inevitably incorporate assessments of value-creating societal outcomes and commercial impact, as well as scientific results (Bozeman & Sarewitz, 2011; Bornmann, 2013; Lane and Bertuzzi, 2011). Therefore, scientists’ grant applications need to signal to reviewers and science boards that their proposed research is relevant to audiences in the funding body’s domain of specialisation, beyond solely science communities (D’Este et al., 2018; Fini et al., 2018).

Again, however, there is a risk that in addressing these criteria on funding body alignment, competing scientists are restricted in the value creation possibilities they can articulate in their grant applications. Supporting this point, Braun (1998) argues that the cognitive development of science is indirectly influenced by a power struggle among the science community, with their thoughts and actions heavily affected by the potential opportunity to acquire “economic capital”. In controlling the material conditions of research production, Braun further argues that funding bodies have a direct influence, not just on what gets researched and by whom, but also in terms of how research practice is structured through the setting of strict procedural criteria related to time spans, milestones and outcomes. Specifically, scientists must “structure their research practice according to the exigencies coming from the administrative logic of the funding agency” (Braun, 1998, p.810).

#### Research team credentials

Team credentials is another signal criteria related to grant funding. According to Lane and Bertuzzi, 2011, p.138), the individual Principal Investigator (PI) is “something of a fiction” with respect to grant success as they are always reliant on a broader group of collaborators. O’Kane et al. (2020) find that a core dimension of the PI role identity is that of “science networker”, in that they form purposeful collaborations with the appropriate experience, status and expertise.

However, scientists need to be cautious when forming and structuring teams as part of their grant application (Laudel, 2006). Funding bodies are reported to penalise and are even biased against, unconventional teams that are considered overly novel or diverse (Banal-Estañol et al., 2019). Exercising caution with respect to the positioning of less experienced scientists on grant applications is another consideration. It is suggested in the literature that funding bodies’ dependency on high-standing scientists means younger less experienced scientists can be disadvantaged in getting their ideas accepted (Braun, 1998; Luukkonen, 2012). Correspondingly, senior or recognisable scientists can be viewed more favourably in the review process. For instance, Banal-Estañol et al. (2019) show how the bias against highly diverse teams is mitigated when the team is led by a prestigious PI. Work by Fong and Wilhite (2017, 2021) draws attention to the use of “false investigators”, namely the inclusion of high-status collaborators on grant proposals without any expectation they will actively contribute to the research, and how their inclusion enhances the prospect of funding success. These dynamics around team formation can have some adverse side effects with respect to the value created through funded research. In discussing their findings on the penalties imposed on structurally diverse teams by funding bodies, Banal-Estanol et al. (2019, p.1838) suggest society misses out as “the resulting reduction of high-impact research, may make it unlikely that the economy will reap significant returns from its investments in R&D”. Perhaps most obvious is the higher concentration of funding arising through the Matthew effect (Merton, 1968). For instance, Sorin and Hannum’s (2014) analysis of the distribution of American Recovery and Reinvestment Act (ARRA) funds by the National Institute of Health (NIH) concluded that the majority of the funds went to PIs who already had non-ARRA NIH grants, resulting in a high concentration of research funding among existing PIs. Bol, de vaan and van de Rijt (2018) also show how funding success increases the chances of accumulating further funding resources in the future. Such developments risk deepening science hierarchies within the science system and exacerbating the level of power and influence highly recognised scientists, networks and research topics hold. A high concentration of research funding can also negatively impact work morale within the science system. Bol et al. (2018) find that one of the reasons funding success for scientists increases the prospects of subsequent funding success is that a proportion of non-winners decide against participating in future opportunities to secure funding.

## Method

This study focuses on New Zealand (NZ) Health Research Council (HRC) funded principal investigators (PIs) as a case of study. Case studies offer an appropriate methodological fit (Edmondson & McManus, 2007) as they allow nascent phenomenon, such as how PIs signal how they have interpreted and addressed funding criteria, to be explored in depth through qualitative insights. As the chief government funder of health research in NZ, the HRC has the vision of “NZ becoming a world leader in high impact, high value health research” (HRC, 2017). To achieve this, the HRC identifies and funds (NZD$126 m per annum as of 2021) research that can “lead to new medicines, breakthroughs and cures, and improves the health and wellbeing of all New Zealanders” (HRC, 2021a).

In general, health research as a discipline is particularly strong within NZ, contributing to more than one-third of all academic outputs (Minister of Science and Innovation and Minister of Health, 2016) and medicine the area in which NZ scholars publish the most highly cited research (RSISPR, 2018). Between 2011 and 2015, field-weighted citation impact for NZ publications in medicine was 1.72, which is significantly above the OECD average of 1.23, and publications in health professions were 1.34 compared with an OECD average of 1.16 (MBIE & MoH, 2017). Consistent with this, along with the Royal Society Marsden Fund, the HRC is one of the two most prestigious and competitive public funders of academic research in NZ. Its system of funding is advanced, well recognised and relevant to a large pool of scientists across all seven NZ universities. HRC-funded research is reported to outperform other NZ funding sectors on quality and impact of publication outputs (HRC, 2016). It is also well recognised and respected internationally, with findings published by HRC-funded teams quoted 44% more than the average for health research publications worldwide (HRC, 2021b). Thus, as both a discipline within NZ and the performance of this discipline by international standards, HRC-funded research offers a worthy case of study to explore how PIs signal how they have interpreted and addressed funding criteria, and some of the possible adverse side effects that can arise from these efforts.

### Data collection and analysis

Data collection was two sided and involved triangulation of secondary and primary sources of data (Gibbert et al., 2008). On the one hand, secondary data from the HRC was collected to understand formal assessment guidelines relating to funding criteria. On the other hand, primary data from successfully funded PIs was collected through semi-structured interviews. This data helped us to understand how PIs as “knowledgeable agents” (Gehman et al., 2018, p.291) constructed and shared their lived experience (Gioia et al., 2013) with respect to interpreting and responding to this funding criteria. To bridge both sides, primary data collection included interviews with PIs who also had experience on the HRC review committees that evaluated grant applications. We offer more details on this data collection and analysis in the sections that follow.

#### Secondary data

A repository of secondary material was gathered and maintained for analysis throughout the research project. This included NZ health policy reports as well as HRC annual reports, application forms and supporting material for researchers including guideline documentation, FAQ sections and video clips. HRC funding is primarily allocated through annual contestable funding rounds with up to 900 application received each year (HRC, 2021c). The present research focused on PI led project, programmes, feasibility studies and emerging research grants.^1^

Managed through the Investment Process Team and its committees, the HRC strive to “run a fair, transparent and robust peer-review process that meets international standards of best practice” (HRC, 2021c). The science assessment engages “around 700 national and international reviewers to ensure we fund research of high quality, led by researchers with the capability to deliver” (HRC, 2021c). Analysis of secondary data showed that scoring in the assessment of project and feasibility applications is based on evaluations of “research rationale”, “design and methods”, “research impact”, “Māori health advancement” and the “expertise and track record of the research team”. For research programmes, the same criteria was utilised along with “cohesiveness of the research programme”. Emerging research grants also used the same criteria; however, track record is replaced by “suitability of the applicant” (HRC, 2021d). Further analysis of secondary data indicated that three criteria appeared most dominant across the various funding types and associated scoring items.

First, research quality and excellence capture the scoring elements of research rationale, design and method and cohesiveness. More precisely, the HRC are committed to funding excellent research which they regard as being “methodologically sound and scientifically robust” (HRC, 2019a, b). Excellence also ensures research “identifies genuine knowledge gaps or needs, and is ethical, well-performed and well-reported” (HRC, 2019a, b). While research excellence within HRC should be original and incorporate “discovery science” (HRC, 2019a, b), PIs are also welcome to build on gains from excellent research carried out nationally or internationally (HRC, 2021b) and encouraged to “focus on what is realistically achievable within your sphere of influence” and to “keep it relevant and keep it credible” (HRC, 2019b). Thus, there is the expectation that funded research will fuel innovative, but feasible, outcomes and lay the groundwork for applied research. More precisely, it is expected to balance “high-risk novel research with the type of research that brings tangible and direct benefits to New Zealanders” (HRC, 2021b). By focusing on excellence, HRC-funded research seeks to “minimise research waste stemming from duplication or unsound results” (HRC, 2019a, b).

Second, the broader mission and impact priorities of the HRC appear to heavily inform the scoring of research rationale, impact and Māori health advancement. As part of the development of the NZ Health Strategy 2017–2027 (MBIE & MoH, 2017), a NZ Health Research Prioritisation Framework (HRC, 2019a, b) was developed to ensure that research efforts (i.e. funders and researchers) are aligned and designed to address areas of greatest need. To this end, in order to contest for HRC funding, applicants must, in addition to pursuing the aforementioned criteria of excellence, address why there research is important for NZ; appropriately account for mana tāngata and advancing Māori health; ensure proposed research has the optimum chance of delivering impact and include measures to improve equity (HRC, 2019a, b). In essence, the HRC expect research applicants to maintain a “clear line of sight” to eventual impact, which in accordance with their mission they define as “a change in individual, societal, economic or environmental wellbeing, beyond contributions to knowledge and skills” (HRC, 2020).

Third, evidence of expertise and track record appears to cut across the various funding opportunities. For instance, with respect to research projects, there is the requirement to provide evidence that the team has the “experience, qualifications and infrastructure to deliver the research” (HRC, 2021e) and achieve the stated outcomes. In the case of research programmes, named investigators are expected to have “extensive” or an “outstanding track record of achievement” (HRC, 2021f). Even in the case of emerging research grants, the “suitability of the applicant” emphasises the “quality of the applicant’s track record” based on publications, awards and prizes (HRC, 2021g). Track record is even emphasised across other scoring criteria for each funding opportunity. For instance in terms of the Māori health advancement scoring item, there is explicit reference to identifying “elements of the team’s track record that provide confidence that this research will optimally contribute to Māori health advancement” (HRC, 2021f). Likewise in terms of the research impact item, there are calls to emphasise how track record of applicants can assist with “knowledge mobilisation” and questions such as “what elements of the team’s track record of knowledge transfer provide confidence in the likelihood of research uptake?” (HRC, 2020).

#### Primary data

Along with ongoing secondary data analysis, we compiled a dataset of all HRC-funded projects, programmes, feasibility studies and emerging research grants over a 2-year period and contacted associated PIs to request an interview. In total, 41 PIs (out of 110 contacted) agreed to participate, with all requesting to have their personal details anonymised. All but two of the PIs were university based, the outliers based in hospitals having recently moved from a university position. Twelve were first time or new PIs and 29 were experienced PIs (i.e. previously and/or currently held a grant). Six of the experienced PIs had also served on review committees for the funder. There were 23 male and 18 female PIs (see Table 1) and the final sample included a range of different funding durations and amounts. All funded research was ongoing at the time of interview, meaning issues related to informant recollection were of little concern. The final sample was suitably diverse and balanced to gather rounded and in-depth insights on how PIs interpret and address funding criteria.

**Table 1:** PI titles and project details (type, duration and value)

		PI details	Project details		
Gender	New/experienced	PI title	Project type	Duration	Project value
Male	E	Dr—Research Leader	Project	36 months	$750,000–$1 m
Female	E	Research Professor	Project	36 months	$1 m–$1.25 m
Male	E	Professor – Research Director	Project	36 months	$1 m–$1.25 m
Male	E	Ass Professor	Project	24 months	$250,000–$500,000
Female	N	Dr – Senior Lecturer	Project	36 months	$1 m–$1.25 m
Male	E	Research Professor	Project	36 months	$750,000–$1 m
Male	E	Professor – Clinical Director	Project	18 months	$550,000–$750,000
Male	E	Research Professor	Project	24 months	$750,000–$1 m
Female	E	Ass Prof—Senior Research Fellow	Project	36 months	$1 m–$1.25 m
Male	N	Dr – Senior Lecturer	Emerging research grant	36 months	$0–$250,000
Male	E	Professor- Research Director	Programme	60 months	$4.25 m–$4.5 m
Male	E	Professor – Research Director	Project	14 months	$250,000–$500,000
Female	N	Dr – Senior research Leader	Feasibility	12 months	$0–$250,000
Female	E	Professor – Research Director	Programme	36 months	$3.5 m–$3.75 m
Female	N	Dr—Lecturer	Emerging research grant	36 months	$0–$250,000
Female	E	Professor – Research Director	Feasibility	12 months	$0–$250,000
Male	N	Dr – Senior Lecturer	Project	36 months	$1 m–$1.25 m
Female	E	Dr – Senior Research Fellow	Project	36 months	$500,000–$750,000
Female	N	Dr – Senior Lecturer	Emerging research grant	36 months	$0–$250,000
Male	N	Dr—Lecturer	Emerging research grant	36 months	$0–$250,000
Male	E	Professor – Research Director	Project	36 months	$1 m–$1.25 m
Female	E	Research Professor	Feasibility	12 months	$0–$250,000
Female	N	Dr—Lecturer	Emerging research grant	36 months	$0–$250,000
Male	E	Professor—Research Director	Programme	60 months	$3.5 m–$3.75 m
Male	E	Research Professor	Feasibility	12 months	$0–$250,000
Male	E	Dr – Senior Research Fellow	Project	60 months	$1 m–$1.25 m
Female	E	Professor- Deputy VC of Research	Project	36 months	$1 m–$1.25 m
Female	N	Dr – Senior Lecturer	Feasibility	12 months	$0–$250,000
Male	E	Professor – Research Director	Programme	60 months	$4.75 m–$5 m
Female	E	Ass Professor/Ass Dean of Research	Feasibility	12 months	$0–$250,000
Male	E	Professor – Research Director	Programme	36 moths	$4 m–$4.25 m
Male	E	Dr – Medical Consultant	Project	36 months	$1 m–$1.25 m
Male	N	Dr – Research Leader	Emerging research grant	32 months	$0–$250,000
Female	E	Dr – Research Leader	Project	30 months	$1 m–$1.25 m
Male	E	Dr – Clinical Specialist	Project	36 months	$1 m–$1.25 m
Male	E	Professor – Research Director	Feasibility	12 months	$0–$250,000
Female	N	Dr – Senior Lecturer	Project	36 months	$1 m–$1.25 m
Male	N	Dr – Senior Research Fellow	Emerging research grant	24 months	$0–$250,000
Female	E	Dr – Research Director	Project	36 months	$1 m–$1.25 m
Male	E	Professor – Research Director	Feasibility	12 months	$0–$250,000
Female	E	Dr – Research Leader	Project	36 months	$1 m–$1.25 m

Semi-structured interviews lasting between 50 and 90 min that incorporated open-ended questions helped to generate the “rich, detailed, and evocative data” (Edmondson & McManus, 2007, p.1162) required to study a novel phenomenon. Seven of the interviews were conducted by phone with the remainder conducted face to face. Case profiles on PIs were prepared and examined from secondary material before interviews, which helped to ensure discussions were more engaging and informative. Interviews broadly focused on (1) competitive funding and careers, (2) research topic/novelty/impact, (3) grant preparation and (4) challenges and critical success factors associated with grant funding. Interesting issues highlighted in preliminary and ongoing note taking were incorporated in subsequent interview discussions. Significant repetition and an absence of new insights within the data after 41 interviews suggested a saturation point had been reached (Strauss & Corbin, 1998). Seven hundred eighty of double-spaced pages of transcripts were created from the data.

Interview data was independently coded by two members of the research team, one of whom had conducted the interviews. Cross-checking and discussions between both coders helped to remove duplicate and unclear codes. This initial stage of coding was exploratory and broadly informed by the primary research questions of the study. Thus, the outcome of this process was the identification of a number of informant-centred labels (Gioia et al., 2013) on how successfully funded PIs interpreted and addressed perceived signal criteria and how this impacted their overall research activities. The quotes associated with these first-order labels were sent back to research participants for confirmation and the opportunity to edit if necessary, which a number of participants did.

In the next stage of the analysis, the two researchers worked together to identify patterns in the data that constituted distinguishable themes. Consistent with Gioia et al. (2013), this process involved progressing from informant-centred labels to researcher informed themes, primarily by incorporating both researchers’ knowledge of the literature as well as the insights they were deriving from their ongoing analysis of the secondary data. Of significant interest to the research team was the extent to which this primary data analysis would confirm and/or expand on the preliminary signal criteria identified through the analysis of secondary data (i.e. research excellence/feasibility, mission alignment, research expertise). In effect, this stage of the analysis resembled an abductive process (Gehman et al., 2018) with key themes (second-order codes) related to perceived signal criteria (i.e. aggregate dimensions) gradually emerging from iterative cycling between initial inductive insights (i.e. first-order codes/labels), secondary data and researcher informed insights and discussions. Of value to discussions among the research team during this iterative process was the fact that one member of the team had relatively recent experience of being a HRC PI and could therefore clarify or explain certain issues that emerged in the data.

**Fig. 2:**
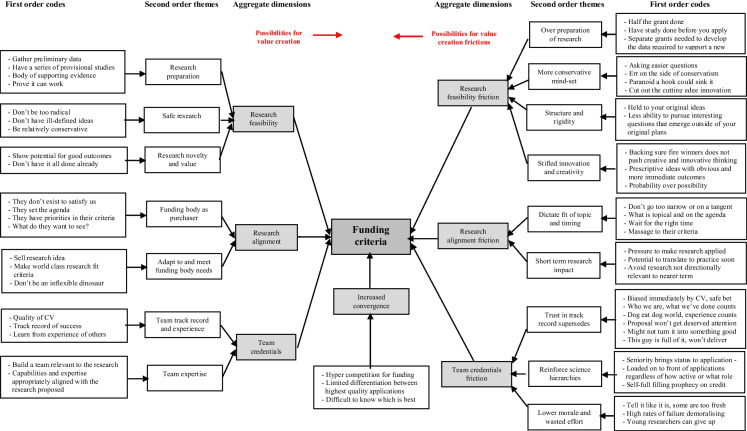
Data structure

What became apparent in latter stages of the data analysis was that data underpinning the themes was distinguishable with respect to how it fed into perceived aggregate dimensions on signal criteria. More precisely, it became clear that while some of the themes were framed positively by PI informants as necessities of addressing signal criteria for funding success, correspondingly there were other themes that were framed more critically by PI informants as undesirable side effects with respect to the implications these had for their overall research agenda. Utilising what Mintzberg (1979) refers to as the essential “creative leap” in inductive research, the research team labelled these two disguisable findings (respectively) as “possibilities for value creation” and “possibilities for value creation frictions” with respect to the outcome of PIs interpreting and addressing perceived criteria when applying for grant funding. Figure 2 presents the complete data structure illustrating how coding of the primary data was undertaken and the resulting findings, that built on the secondary data analysis, were organised. In the next section, we present evidence to support these findings.

## Findings

Our findings show that scientists address three criteria signalled by the funding body—research feasibility, research alignment and team credentials—when preparing grant applications. Effectively addressing these criteria helps to make their proposal competitive, improving their prospects of funding success and value creation from publicly funded research. However, due to the high levels of competition for grant funding, scientists’ search for a differentiating edge can lead to them over-addressing or -conforming to these criteria. This in turn can result in some adverse side effects or frictions for value creation. We expand on these findings below.

### Funding criteria and value creation

Crafting and submitting research grants is a challenging exercise for scientists. Our findings provide evidence of three critical requirements that scientists first sense/interpret and then address when preparing their grant proposals. These requirements are demonstrating the proposed research activities are *feasible* and sufficiently *aligned* with the programme criteria set out by the funder and that the *team credentials* of the science team led by PI are suitable. Our findings indicate that all three requirements must be effectively addressed by scientists. This point is illustrated through the following insights provided by three of our research informants.

In the first comment, in reflecting on how she led several successful research grants, the research informant details how she was always careful to address all three requirements (note: content in italics added by the authors for emphasis).

When it comes to actually putting together a grant application, I know that I’ve got to have a series of studies (*research feasibility*), I’ve got to show my capacity and ability to do it (*team credentials*) and I have to fit in with the priorities articulated (*research alignment*), we make sure we cover all of the bases. (The) process is good for forcing those disciplines.

In contrast, in the second comment, another research informant who also served as a reviewer on a number of funding body panels explains how certain grant submissions are less likely to be successful if they omit or inadequately address one or more of these three requirements identified.

I’ve been on a quite a few grant reviewing committees and you know the top ones by more experienced teams really know how to write (a) successful application…(others) they just don’t have the CV (*team credentials*) and the writing and selling abilities (*research feasibility and alignment*) that the more experience ones have.

The third comment from another informant further reinforces this point, highlighting how failing to address even one of the criteria (in this instance team credentials) can be fatal to the competitiveness of a grant application. Further detail and insight on these criteria are provided in the paragraphs that follow and support with some illustrative evidence in Table 2.

If you don’t have a track record your chances of getting funded are miniscule, there’s almost no opportunity. They (the funder) are going more and more that way with track records becoming a bigger part of the funding process.

**Table 2:** Research grant criteria

Funding criteria	Illustrative data
Research feasibility Is there enough evidence provided to prove the proposed research can be done?	“You must gather as much preliminary data as possible to show that your ideas are actually solid without actually having done it all beforehand, but enough to convince the referees that your idea is a winner. We’re all capable of generating great ideas but with no preliminary data there’s no chance of getting funding” “You don’t fly at HRC with radical ideas or zany stuff. You just don’t do that. For our project, we proved that it could work in our environment and we provided some initial results. That is what you need for the HRC. The HRC need to know that this is going to work and this is going to provide results” “You really need to do the preliminary work in the first instance before even applying for funding like HRC. They want to see a body of evidence supporting the question you’re putting forward and then to say ‘right this is what we have found and this is what we’re proposing to do’”
Research alignment Does the proposed research sufficiently align with the priorities of the funding body?	“The reality is that funding bodies don’t exist for our beck and call or to adapt to what our interests are. They define the agenda because they’re the purchasers. I think you can lobby them or try and persuade them to see that a different approach is of relevance and is necessary to fund but you can’t be an inflexible dinosaur. You have to position yourself” “For our studies we start off with an original concept and then we make it fit into the criteria for the funding agencies so we have a whole bunch of concepts and ideas and then we go down the list and decide okay, which of these is most fundable in meeting the HRC criteria”
Team credentials Does the team hold appropriate expertise, track record and experience?	“Important for me is ensuring that you’ve got the expertise because the referees first and foremost want to see that the people have considered the issues and they can actually do the project – ‘have they got enough fire power in their application?’” “I realised that getting a good team was critical to getting a grant (so) I pulled in the people that I knew were successful that might want to be involved but also were relevant. I was comfortable that I was the right person to lead it but obviously I was asking all these experienced people to let me bounce ideas off them if needed and that’s pretty much what we did”

In terms of research feasibility, informants unanimously explained that they are careful to demonstrate how the research they are proposing is novel but achievable and therefore reasonable to fund. According to research informants, limited funding availability means funding bodies are likely to be more conservative in how they allocate grants. Such caution in turn demands that submitting scientists make every effort to convince funding body review committees that their proposed research is doable, namely that it can be a “winner” in terms of producing achievable and value-creating research outcomes. As commented by one informant:

If you think of the world we live in, funding is so tight…the HRC are probably hedging their bets so if they can find a research project which is relatively conservative but has the potential for very good outcomes, they’re more likely to fund that.

Cognisant that funding bodies hold this view, our findings provide evidence that submitting scientists go to great lengths to “prove” their research is viable and therefore worth backing—“you have to be secure and convincing in the feasibility and the likely outcome”. The inclusion of preliminary data and initial results relating to the research questions posed provides greater confidence to grant review panels that the research can be completed successfully.

Research informants also detailed how considerable effort is directed towards ensuring their research ideas are adapted to sufficiently align with the expectations and priorities of the funding body. In recognising that funding bodies essentially represent the customer, our informants explained how they needed to filter out various research ideas and position their research activities in such a way that they could credibly contribute to the agenda of the funding body, rather than vice versa. As illustrated in the following comment from a research informant, there is a dual pressure on research scientists to, on the one hand, undertake leading edge and novel research and, on the other hand, ensure their proposed research is appropriately aligned with the needs of the funding body:

Speaking personally, we go ahead and we do the research that we think is world breaking and advancing in our field…we carefully mould what we’re doing into what the funding body would want to see.

A third requirement or criteria associated with preparing a grant proposal relates to forming collaborative teams with the appropriate credentials to successfully complete the research on time and within budget. Our findings provide evidence that submitting scientists are aware of the importance of forming science teams that will be perceived favourably with respect to the expertise and track record they hold. Appropriate expertise is important as it gives greater confidence that the team will be able to address the stated research problem. Evidence of research productivity and funding experience are also important as they demonstrate a track record of success and of producing research outcomes from funding, as well as a mechanism through which less experienced team members can learn from more experienced peers.

### Value creation frictions: adverse side effects

Although effectively addressing these funding criteria helps scientists to make their proposal more competitive, which in turn improves the prospects of funding success and value creation, it also poses some potential downsides. More precisely, as the criteria become increasingly recognised among grant-seeking scientists and accepted as essential to address appropriately, our findings indicate it is difficult for scientists to deviate away from these. This results in greater convergence across grant applications and greater difficulty for reviewers to distinguish among the top percentile in their evaluations. This point is exemplified in the following comments from two research informants who served a number of times on the funding body review panel.

We’ve got a situation that is so competitive now that only six per cent of the grants get funded. We are actually getting to the point where it’s probably impossible to pick whose grant is better. At least 25 per cent of the grants are very good and worthy of funding so the ones getting through aren’t necessarily the best.

I don’t think we can know for sure if it is the very, very best that is getting funded because once your funding rates go down below about 20 per cent, you can’t distinguish amongst the very top you’re just picking amongst the good. If the question were, ‘is this application in the top 25 per cent?’ grant reviews would be must less inconsistent. But because the question is, ‘is this application in the top 5 per cent?’ peer review panels simply can’t distinguish that.

Our findings provide evidence that, in anticipation of such levels of competition and complex deliberations among reviewers, scientists’ efforts to signal a differentiating edge in addressing these criteria can result in them over-conforming to the signal. When scientists over-address or -conform to the perceived funding criteria, it can result in some corresponding frictions for value creation as an adverse side effect from their efforts. We next detail these value creation frictions.

#### Friction 1: Research originality-feasibility

In referring to the need to prove the feasibility of their proposed research, a number of informants explained that it was possible they could over-reach in addressing the perceived expectation around providing sufficient pilot data and preliminary evidence. For instance, as illustrated in the following comments, a number of informants who were successful with their grant submission felt they had much of the research completed before submitting it as a proposal, such was the perceived pressure to validate their chosen line of inquiry:

We played the game. You’re basically telling people in your grant what you’re going to do, you practically have to have half the grant done before you’re going to get the money for it.

My impression is that the HRC probably aren’t going to fund something unless it really has some firm evidence or likelihood of working. But then there is that line, and no-one knows quite where it is, about how much pilot data you need. In some instances it’s almost like you need so much pilot data that you’ve almost done the study before you’ve even submitted the application.

While this could limit opportunities for scientists to explore new or distant areas within the research they proposed, it also tended to slow down the research process as it limited their capacity to efficiently pursue research opportunities they identified. This view is evident in the following remarks from a research informant.

You need some good data, something tangible, before you can even go to HRC. They won’t look at you otherwise. So I was thinking, we have a really cool idea but we’ve got no data so we have to get another grant first to start that process.

Research informants also explained that the research they chose to submit for grant funding tended to be safer or less risky. Scientists indicated that one of the reasons they adopted such an approach was the heightened competition for funding—“funding is now so competitive it is a bit more conservative with the questions you ask probably a bit easier to answer”. While “easier” might be a slightly misleading view on the type of research that gets funded, a large number of research informants did point out that a core challenge they encountered in preparing their grant applications was blending the novelty of their research with some concrete assurances around predictable research outcomes.

It’s got to be little bit novel to be funded, and not a ground-breaking paradigm shifting proposal. It is a delicate balance, but I think we err on the side of excessive conservatism. For me being too prescriptive and only backing ideas that have immediate and obvious benefits is not always the best approach.

Our findings provide evidence that these impressions among the science community have several potentially adverse implications for value creation. In terms of individual scientists, we find that scientists can become more conservative in their approach, to the point that they are almost paranoid to take minor risks that could scupper their chances of funding success. Moreover, if among the perceived critical criteria for funding success are precision and certainty around research plans and outcomes, this can propagate approaches and mindsets among scientists that are in line with these expectations. These findings are illustrated in the following comments from two research informants:

I still inherently write quite conservatively because the nature of how competitive funding is means that actually anyone who finds a hook, even it is just mentioned in passing, that hook can sink you and so conservatism still reigns in my mind.

I appreciate the importance of having prepared mind and research discovery, but you know…the innovative process is not something that is promoted. Backing sure fire winners does not promote new or creative thinking.

Several research informants also explained how there was increasingly less scope to explore emergent lines of inquiry that arose over the course of the funded research. Instead, there was a sense among research informants that, once funded, there was an expectation the research questions and milestones set out in the original grant proposal would be adhered to, regardless of what intriguing or high potential questions emerged. As stated by one respondent, “it doesn’t leave much room to explore new areas so it is a wee bit too prescribed”. This level of rigidity not only delayed or prevented valuable avenues of research from being explored, but it also frustrated researchers by binding them to pre-prescribed lines of inquiry, even if they were more intrigued by others. The following comment illustrates this point:

A long time ago, it was quite possible to write a grant with all your aims and then never address them. As you get into the work other questions would come up which appeared more interesting to answer than the first lot so you’d just answer them and as long as you were publishing, it was fine. But now you are held to what your original ideas were, it is all very prescriptive.

Beyond indoctrinating a more cautious and rigid approach among individual scientists, insights provided by research informants indicate that excessive prioritisation of, and conformance to, certain requirements such as feasibility can potentially deprive the wider science system, and society more generally, of novel ground-breaking discoveries. More precisely, several research informants commented that some important innovative discoveries are most likely lost within a system that is uncomfortable funding outside possibilities, preferring instead ideas with a high likelihood of “probable” success. Consequently, scientists submitting research grants will often deliberately “cut” out high-risk components of the proposed research project to improve their chances of funding success; however, this can also lessen the probability of some “cutting-edge innovation” emerging from the funded research. Support for these findings are evident in the following comments.

There is a real problem for research with funders. (They) will fund a ‘probable idea’, this is in contrast to a ‘possible’ idea. So funders will come in behind an idea that’s got proof of principle, the prototype, the preliminary data, and there’s strong support for this being successful and translating through to something that’s going to help…but this leaves a huge gap between the ‘possible’ idea and the ‘probable’ idea. We lack funding for the former, and this is stifling for (our) innovative research programs.

I think they lose some real cutting-edge innovation because you have to cut much of that type of stuff out. I mean the most exciting projects I am doing at the moment…(are) totally new but HRC would never have touched it with a barge pole. I didn’t even try it. I knew they wouldn’t.

#### Friction 2: Research alignment-autonomy

A second value-creating friction uncovered relates to the risk that scientists over-reach in their efforts to have their proposed research conform to the agenda and priorities of the funding body. Our findings provide evidence that when this occurs, research autonomy is somewhat strained as researchers’ efforts to align with funding body expectations influence what they choose to study and how they set about structuring their research programme. In terms of influencing what to study, several research informants explained that they continuously surveyed funding signals to keep updated on what was topical and what areas were most likely to get favourable attention and funding. As illustrated in the following comments from a research respondent, choosing topics that could be perceived as obscure, regardless of whether they were a primary area of research interest and expertise for scientists, was not wise as the resulting research outcomes were likely to be perceived as equally narrow and therefore unappealing to public funders in terms of impact and societal return on investment.

You don’t want to position yourself in a niche with some rare disease. How do you justify spending the research health dollar on a small population? You position yourself where the funding is. It’s about catching the sexy topic. What’s sexy at the moment and then put yourself there.

In other instances, research informants explained how, in their efforts to adapt their proposed research topics and to have them align with the mandate and priorities of the funder, what was eventually funded was more in line with what the funding body wanted as a research project than the actual scientist who submitted the grants. This point is illustrated in the following comment from a research informant.

A lot of that was to some extent deliberately massaging our grant, we did the study that their criteria wanted, it wasn’t possibly the study we would’ve pursued if we’d just stayed on our own. But I think you’ve got to play the game. You just can’t go off on your own tangent.

The research alignment-autonomy friction is also informed by sensing the timeliness of grant applications on particular topics. For example, research areas that may be prominent in certain geographical areas or for specific populations may not be as topical in/for others. This is particularly significant concerning the international mobility of research scientists, as their research programmes may be disrupted and delayed if they are not perceived to be as relevant or topical within their new professional environment. The comment from a research informant supports this point.

I have an international reputation in the field of XXX for work that I started when I was in the US. The subject wouldn’t have scored anything here when I came back to New Zealand because it just wasn’t on the agenda here, it wasn’t until I was back for five years that I felt the time was becoming right to start a study in New Zealand.

Further evidence on how research alignment can strain researcher autonomy relates to how, or over what time, scientists propose they will undertake their research and produce research outcomes. To have the best chance of success with grant submissions, scientists report they feel it is important to provide some assurances they will produce research outcomes that will be reasonably easy to translate into practice within the nearer term. Illustrating how these perceptions influence research practice, one informant explained how, after several grant applications on a particular project, they were eventually funded, but this required them to “appropriately” incorporate previous feedback and make their research project more applied than they would like. Another explained how they deliberately decided against submitting grant applications relating to more basic or longer-term research agendas as they sensed these would have little likelihood of success. Comments on both of these illustrative examples on research alignment are provided here.

This whole programme that we’re undertaking in a way was developed under perceived pressure to take our basic research stream to a more applied area where it would be more likely to be funded. We got subtle and not so subtle hints. So the whole thing has shifted as a general topic for me.

Funders can be a bit short sighted. They’re essentially looking at stuff that can translate directly into practice benefits in the shorter-term, say two years. But the reality is that a lot of research has to be built up over a longer time. Like we do some bio-mechanical things that looks at the way the body moves, science that will inform stuff later down the track but I doubt we would get funding for that because it’s not directionally relevant to now. I think they need to adjust their balance a bit there.

#### Friction 3: Team credentials-science hierarchies

A third friction uncovered in our analysis relates to the potential counterproductive consequences of scientists over-addressing the issue of team credentials. A large number of informants offered the opinion that there was low trust when it came to grant applications led by PIs or teams with underdeveloped track records. It was the understanding of research informants that these applications introduced higher (and unwelcome) risk to the decision-making of funding body review panels. More precisely, there was a clear sense among informants that applications were screened according to the credentials and funding experience of the PI and their team, more so than the merits of the research idea being proposed. These perceptions increase the likelihood that novel and potentially valuable ideas are overlooked in the review process for being too new or risky or are not even submitted at all in full expectation that they would be overlooked. The following comments from two research informants illustrate these findings.

Without any track record the committee may not take it because they might think this guy is full of it and doesn’t really know it. They’ll say ‘without going into literature in any more depth, I just can’t be absolutely certain he’s got it right and we don’t know if he can actually deliver, we haven’t seen that before so maybe it’s a bit more high-risk.

I think there are some process issues. For example, in the EOI stage if you’re on the panel you maybe have 100 EOIs to read, what are you going to read? You’re going to read the summary and you’re going to look at the CVs and you’re not likely to spend half an hour thinking ‘mmm, that’s a really good idea, that’s got potential’. So you know you’re going to be biased immediately by the fact that this guy has never got a grant whereas this guy is a Professor with HRC grants for 20 years so he’s a safe bet.

These findings are further supported by comments from other respondents. For instance, one respondent who has also served on several funding body review panels confirmed that the research ideas of less experienced PIs and teams, regardless of their merit, can encounter some additional challenges in the review process. Insights provided by another research informant would suggest they have little sympathy for less experienced scientists in this regard, as they have other funding opportunities and avenues to develop their careers. It is likely that experienced PIs such as this particular respondent feel like that because they had to contend with such challenging circumstances in their career. These respective comments are provided here.

In the selection process the importance of the credibility and track record is huge. If you’re just an emerging researcher or you just have a really good idea you will be disadvantaged. I have seen reviewer comments that clearly state the PI or their team is not experienced enough.

I think emerging researchers need to understand that it is a bit like anything in the world. It’s a pyramid. There isn’t room for everyone at the top, it is not a utopian society, it’s a dog-eat-dog world. You know these people get a lot of internal funding and there are lots of starter grants and smaller grants to compete for. They can target these with their first few grants, prove a track record and move up the ladder.

These circumstances have consequences beyond potentially missing out on novel research ideas that could lead to innovative breakthroughs. They may correspondingly result in research ideas that arguably hold less potential value getting resourced. This point is clearly apparent in the following comments from two research informants. In the first, the respondent shares the view that name recognition and seniority is correlated with higher levels of trust and perceptions of a safe pair of hands. In the second comment, the respondent offers insights on their experience that would support this view, namely that recognition and track record trump, or even compensate for, quality of research idea and grant application.

With a more senior investigator, you kind of get trusted more. They think ‘we know that name, he knows what he is doing.

I mean two of the projects we’ve had recently actually weren’t evaluated or scored terribly well and they were funded. So part of that has got to be who we are and what we have done.

Again, comments provided by a respondent serving on a funding review panel offer further support for this finding. In these remarks, which are provided below, they explain how in review discussions, an application and idea that are evaluated as “really good” might still end up losing out as the panel feel more reassured funding someone more experienced who will (more likely than not) produce “something good”. Thus again, there is the dual risk that potentially valuable ideas will miss out and safer possibly inferior ideas will win out, just because they provide more certainty around research outcomes based on the credentials of the PI and the supporting team.

During a review we had discussions around really good applications but one of the criticisms was ‘this young guy doesn’t have much of a track record and might never turn it into anything good’. Some argued that you have to give a chance for such a good application. The other argument was, would it be better to give the $1.2 m to this Prof who will do something good with it regardless?

Another potentially counterproductive consequence of these perceived expectations among the science community is that there can be some gamesmanship leading to a distortion of credit. More specifically, our findings provide evidence that scientists are acutely aware of the degree to which records of accomplishment of team PIs influence the impressions and evaluations of review panels. As a direct consequence of this, there is pragmatic, although uncomfortable, realisation among the grant submitting scientists that more experienced or high-status scientists should be the leading figure on grant applications, regardless of how involved they actually are in formulating the application or to what degree they will realistically lead the workload should the grant be successful. These findings are illustrated below by comments from both experienced and less experienced research informant scientists.

I like to be on an application because of what I can bring. But also some of my colleagues here and I are at a level of seniority where we realise that when we go on as the PI we bring status but if we put somebody junior as the PI even if they lead the process the grant won’t get the attention it deserves so it’s a challenging balance that has to be managed.

There seems to be a tendency for the process to get hidebound in terms of track record…where people get loaded onto applications, sometimes at the front, and then you know the (less experienced) person who is second or third down the line is the person actually doing it. It becomes a self-fulfilling prophecy where the person who is getting a better track record going for another application is sitting on top but not doing as much or not necessarily having written the initial application.

As explicitly referenced in the latter comment, a potential outcome of such deliberate positioning by scientists is a distortion of credit. More precisely, while those who already have a higher level of status and a more developed track record can be leveraged to increase the likelihood of success, when this occurs the disparity in status is increased further. Taken together, these findings on managing the ordering of scientists on grant applications along with the earlier findings relating to how preference can be given to safer research ideas led by more “secure” applicants runs the risk of reinforcing scientific hierarchies.

Related to the maintenance and entrenchment of existing science hierarchies is the potential lowering of morale and career exits among emerging scientists. Likely under immediate and continuous pressure within their institutions to acquire external funding, many of these scientists spend an inordinate amount of time writing grants with a very limited chance of actual success. As explained in the following comments from two informants who serve on funding review panels, although the likelihood of being unsuccessful is in reality very high, the failures, particularly when they mount up, can be devastating and difficult to process with some deciding to pursue careers outside of academic science.

They should rather than telling the whole world ‘you’ve got as good a chance as anyone of getting it’ be explicit and tell people if they are too fresh and don’t have the skills unless they’re part of a high producing team. I sat on a grant assessing committee last year with 93 grant applications. We made six awards. Now those 93 groups of people all thought they had a chance at getting the money. These people will be devastated when they fail, they are very bright people who are not used to failure and it demoralises them.

Some young researchers give up on the career. It can be completely demoralising for them. Some comments are very personal and these poor guys get completely chopped up in terms of, not the study design, but their research ability.

## Discussion

Our research offers new insights on how grant-seeking scientists interpret and address funding criteria. It also provides novel insights on how the two-way interaction between the science community and funding bodies in the competition for limited funding can, on the hand, create value, but, on the other, result in frictions that curtail value creation. In this section, we present the main contributions arising from these findings. First, we discuss the funding criteria identified in our research and how addressing these has implications for the value created from public grant funding. Second, we discuss the implications of our research findings for signal theory, in particular the new insights our research provides on the cognitive processes of signal actors.

Our research identifies a portfolio of criteria that underpin value creation from public grant funding. Specifically, our findings provide evidence that successfully funded scientists simultaneously address (1) research feasibility that there is enough evidence provided to prove the proposed research can be done, (2) research alignment that the proposed research sufficiently aligns with the priorities of the funding body and (3) team credentials that the team demonstrates appropriate research expertise for the research problem in question, as well as suitable track record and career experience. Furthermore, our research proposes (see Table 3 for a summary) that the extent to which scientists address, either appropriately or excessively, all or some of these criteria will impact possibilities for value creation and value creation frictions from public grant funding.

**Table 3:** Funding criteria and possibilities for value creation and value creation frictions

Scientist	Criteria signalled by funding body			Value creation possibilities	Value creation friction possibilities
Addressed	Feasibility	Alignment	Team	Addressed appropriately	Over-addressed
All	√	√	√	• Enhance scientists’ prospects of funding success • Assists funding body evaluations • Funding of high-quality research that is doable, valuable and which grows scientific human capital	• All of below
Only two addressed	√	√	×	• Proposed research is feasible and aligned with funding body priorities • However, little chance of funding success as the appropriate track record, key expertise and/or funding experience is lacking within the team	• Probability trumps possibility • Prescriptiveness over flexible exploration • Conservatively thorough over opportunistically agile • Institutional pull moulds scientist push • Less control of what to study when • Prioritisation of nearer-term translation and impact
	√	×	√	• Proposed research is feasible and the appropriate track record, expertise and experience are apparent within team • However, little chance of funding success as the proposed research is not sufficiently aligned with funding body priorities	• Probability trumps possibility • Prescriptiveness over flexible exploration • Conservatively thorough over opportunistically agile • Team/idea security trumps idea true potential • Science hierarchies leveraged and reinforced • Increased inefficiencies in effort • Decreased morale among less experienced scientists
	×	√	√	• Proposed research is aligned with funding body priorities and the appropriate track record, expertise and experience is apparent within team • However, little chance of funding success as the proposed research is not sufficiently feasible	• Institutional pull moulds scientist push • Less control of what to study when • Prioritisation of nearer-term translation and impact • Team/idea security trumps idea true potential • Science hierarchies leveraged and reinforced • Increased inefficiencies in effort • Decreased morale among less experienced scientists
Only one addressed	√	×	×	• Proposed research is feasible • However, little if any chance of funding success as proposed research is not sufficiently aligned with funding body priorities and the appropriate track record, key expertise and/or funding experience are lacking within team	• Probability trumps possibility • Prescriptiveness over flexible exploration • Conservatively thorough over opportunistically agile
	×	√	×	• Proposed research is aligned with funding body priorities • However, little if any chance of funding success as proposed research is not sufficiently credible and the appropriate track record, expertise and experience is lacking within team	• Institutional pull moulds scientist push • Less control of what to study when • Prioritisation of nearer-term translation and impact
	×	×	√	• The appropriate track record, expertise and experience are apparent within the team • However, little if any chance of funding success as proposed research is not sufficiently credible or aligned with funding body priorities	• Team/idea security trumps idea true potential • Science hierarchies leveraged and reinforced • Increased inefficiencies in effort • Decreased morale among less experienced scientists

More precisely, our research shows that when grant-submitting scientists effectively address these funding criteria it enhances the competitiveness of their grant proposals and their prospects of funding success. It also enhances value creation possibilities from public grant funding through the submission and funding of higher quality research projects and programmes that are doable, valuable and which sustain and grow scientific human capital. When one or more of these criteria is not addressed, associated grant applications are likely to be less competitive, and the prospects of funding success and value creation are diminished. Furthermore, our research indicates that over-addressing one or more of these criteria can be counterproductive for value creation. For instance, scientists that over-address on the feasibility criterion are likely to put forward more conservative lines of inquiry that prioritise offering assurances around particular research outcomes. They emphasise probability and predictability over possibility. The research processes they propose will be more thoroughly prepared, prescribed and structured with less scope or motivation to adapt, and explore new and exciting avenues of inquiry that emerge over the course of the research. Over-addressing on the research alignment criterion pushes grant-submitting scientists to mould and fit their research, both in terms of topic and timing, to the expectations and priorities of funding bodies, more so than shaping scientific trajectories through their own unfiltered research ideas and objectives. Scientists that over-address this criterion will also prioritise the preparation of research proposals that credibly demonstrate a translation towards application and practice in the nearer term. Finally, to ensure perceived deficiencies in team capability and experience do not emerge, grant-submitting scientists that over-address the team credentials criterion exploit, and therefore reinforce, science hierarchies. They take extensive measures to ensure the team formed to undertake the proposed research is evaluated positively and not penalised for lacking firepower and scholarly standing. This can disadvantage the development of, and lower morale among, emerging scholars and also result in the formation of “safer” teams being prioritised over the quality of research ideas, particularly when these ideas are associated with less experienced teams.

These findings make a number of important contributions to the literature on science funding. First, recent literature has highlighted the value that can be created from participating in the competition for grant funding (Ayoubi et al., 2019). Our research adds to this perspective, showing how resourcefulness behaviours at the individual-level (Michaelis et al., 2022), that is the actions of scientists, are at the core of value creation from public grant funding. Our research provides new insights on how resourcefulness behaviours are fundamental to the two-way signalling interaction between members of the science community, who prepare grant proposals based on how they interpret and address funding criteria, and funding bodies, who signal and evaluate against these criteria. In essence, our research shows that scientists look to address funding criteria in differentiated ways which can enhance value creation through better resource deployment. Second, our research makes an important distinction between the possibilities for value creation and the value creation frictions that can unintentionally arise in the pursuit of science grant funding. Specifically, we offer new insights on how the practices of research scientists competing for grant funding can produce unintended side effects (Scholten et al., 2021). Our research shows that, with limited opportunity to deviate from the criteria signalled by the funding body, grant-submitting scientists’ efforts to address the criteria “most” effectively leads to over-conformance, and this gives rise to some undesirable value creation frictions.

**Fig. 3:**
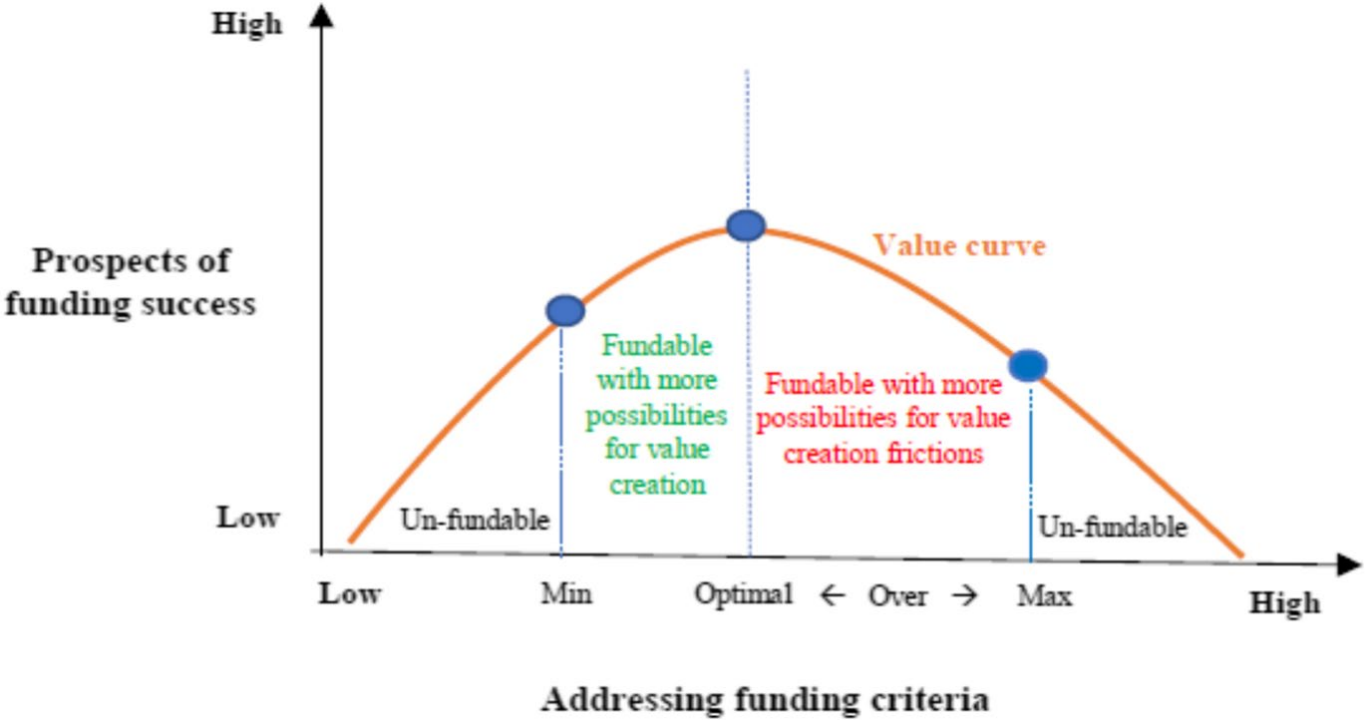
Possibilities for value creation and value creation frictions from science funding

In adopting a signalling perspective on grant funding, our research also makes a number of contributions to signalling theory (Connelly et al., 2011; Spence, 1978). First, by considering the two-way interaction between scientists and funding bodies, we address calls to pay closer attention to the cognitive processes of signal actors (Drover et al., 2018). Researchers have emphasised that signal receivers vary in the way they interpret and allocate attention to multiple signals (Drover et al., 2018); however, our research advances these insights by examining a unique process involving an ongoing and interdependent exchange of signals between actors who each adopt the position of signal sender and receiver. Within this process, we offer new and in-depth insights on how scientists differentially interpret and address the multiple criteria signalled by funding bodies in the competition for grant funding. Second, our research provides new insights on both the value creation and value creation frictions that can arise through the signalling process. Specifically, our findings highlight that when a signal environment is highly competitive, or noisy (Steigenberger & Wilhelm, 2018), and the same signal criteria can be interpreted and addressed in different ways, efforts to acquire the signal can produce (the undesirable side effects of) frictions for value creation, as well as value creation. The value curve presented in Fig. 3 illustrates this proposed contribution.

In essence, based on our findings, we propose that the overall value created through public grant funding in science is determined by the extent to which the funding criteria is addressed in grant applications. More precisely, in line with our first stated contribution to signal theory above, the value curve in Fig. 3 illustrates the level of scope for grant-submitting scientists to differentially address the multiple criteria signalled by funding bodies, and how this in turn impacts the prospects for funding success. The “unfundable” portions of the curve reflect instances in which scientists (1) inadequately address or omit one or more of the funding criteria within their grant proposals or (2) inappropriately over-address one or more of the funding criteria. For instance, proposing research that is considered unoriginal or with unrealistic or uncoordinated team credentials. In contrast, the area within the min–max range of the value curve reflects instances in which grant-submitting scientists have addressed all funding criterial to differentially acceptable levels and therefore have better prospects of funding success. Furthermore, and consistent with our second stated contribution to signal theory above, the value curve also illustrates varying possibilities for value creation and value creation frictions based on the level of effectiveness with which scientists address the funding criteria. Again, within the unfundable areas, the criteria have not been appropriately addressed; therefore, the possibilities for value creation and value creation frictions are low and high respectively. However, within the min-optimal range, the funding criteria have been appropriately addressed to varying degrees of effectiveness, resulting in corresponding increasing possibilities for value creation. Within the optimal-max area, while the criteria have still been addressed effectively, due to the hyper-competitive or noisy (Steigenberger & Wilhelm, 2018) funding environment, one or more of the criteria has been addressed more than is optimum, and this we propose results in the emergence of unfortunate value creation frictions that detract from the overall value created from a grant funding programme. The more the funding criteria is over-addressed, the stronger the value creation frictions that arise until such point that grant applications are deemed unfundable, as explained above. We believe these insights represent an important contribution as they emphasise the opportunity cost for overall value creation associated with grant funding. Failing to elucidate and appropriately recognise value creation frictions means the value creation possibilities from publicly funded science are only partially understood.

Finally with the growing empirical focus on the evolution of entrepreneurial and innovation ecosystems (Cantner et al., 2021; Cho et al., 2022; Mack & Mayer, 2016; Rocha & Audretsch, 2022), our study offers a micro-level perspective that researchers can further extend in their attempts to address a fundamental question of how do entrepreneurial and innovation ecosystems evolve. Given the role public science and public sector entrepreneurship programmes play in the formation and evolution of entrepreneurial and innovation ecosystems (see Link, 2022; Hayter et al., 2018; Leyden & Link, 2015), there is a necessity for a micro-level understanding of the attitudes, behaviours, activities and actions of scientists. This can further enhance our current understanding the evolution and dynamics of entrepreneurial and innovation systems. In particular scientists in the principal investigator role (Mangematin et al., 2014; Del Giudice et al., 2017; Modic & Yoshioka-Kobayashi, 2020). They have some positional power within an ecosystem through the securing public, private or philanthropy funding to advance and apply knowledge that can have potential value creation benefits for ecosystem actors (Cunningham et al., 2018). Moreover our study highlights the need to better under the role of funding agencies in how they support the evolution of entrepreneurial and innovation ecosystems.

## Conclusion

In this research, we argue that public funded grants in science represent valuable signals and frame scientists and funding bodies as both senders and receivers in an ongoing two-way signalling interaction. Within this context, we examined the process of signal acquisition or the optimal attributes put forward by grant applicants to maximise their chances of funding success. Specifically, we addressed two questions (1) how do scientists signal how they have interpreted and addressed the criteria set by the funding body? and (2) what are some of the adverse side effects that can arise from scientists’ competitive efforts to address this criteria? Our findings show that effectively addressing three signal criteria—research feasibility, research alignment and team credentials—enhances the competitiveness of scientists’ grant proposals and this in turn improves prospects of funding success and value creation. However, our research also shows that scientists can over-address these criteria in the hyper-competition for funding, which can be counterproductive for value creation. More precisely, such over-conformance can produce undesirable side effects in the form of value creation frictions. Our research therefore makes an important distinction between the possibilities for value creation and the value creation frictions that can unintentionally arise, depending on how scientists pursuing grant funding interpret and address the criteria signalled by the funding body. In essence, our research shows how the aggregate level of value created through publicly funded research is determined by the interconnected sending and receiving or interpretation and evaluation of signal criteria between scientists and funding bodies.

Our study has some practical implications. Foremost among these are the potential societal implications associated with the value creation frictions uncovered in our research. For instance, communities may miss out on important ground-breaking discoveries and innovations as a result of novel or longer-term research ideas being blunted (i.e. overly prepared and structured) or overlooked (i.e. if lacking necessary team credentials) in favour of safer and more secure, but not necessarily better, research proposals. Also, science as a career may become less attractive to existing and emerging scientists as they become increasingly disillusioned and frustrated. This may stifle the overall quality and quantity of research taking place, which may in turn affect societal advancement and economic development given how important research-based innovation activities are for knowledge-based economies. In addition to these societal implications, policymakers at the macro-level need to carefully consider how they set and implement the signal criteria, novelty thresholds, norms and expectations for the funding agencies and scientists. Ultimately, the key determinant of value creation in the science system (with respect to grant funding) is how effectively scientists address signal criteria at the micro-level; therefore, it is essential that policymakers are doing enough to effectively shape and communicate the signal criteria they want to see addressed. Related to this, they need to ensure they provide enough resources dedicated to supporting novel, early stage and higher risk research, either within or separate to funding lines with lower novelty thresholds. More generally, given the funding constraints and high levels of competition associated with grant funding, policymakers need to decide if the trade-offs on potential value creation are worth it or whether it is time to explore new ways to organise the science system with respect to grant funding in ways that can continue to harness the best teams and ideas but with less value creation frictions as a by-product.

For funding bodies, our study illustrates that the evaluation and selection system need to be transparent and itself open to evaluation. Funding bodies need to consider carefully the selection of evaluators and monitor the consistency of their decisions across funding calls. Moreover, funding bodies may need to consider additional evaluative approaches where there is a minimal distinctive separation between funding proposals. This may require a more fundamental rethink of the evaluation criteria and weightings and how evaluators are selected and inducted by public funding bodies. A further consideration that arises from our study for public funding agencies is how they account for equality and diversity and supporting scientists at all career stages. Specifically funding agencies need to consider what practical supports and schemes can be targeted at scientists at the early and mid-career stages, and how they might better identify and eventually eradicate the false investigator phenomena through their evaluation processes. For universities, a consideration from our study is how they can best support scientists in situations where they have been repeatedly unsuccessful with grant applications. For instance, universities might wish to consider putting in place formal support systems for scientists to repurpose failed grant applications for alternative opportunities and support structures dedicated to building individual researcher and research team resilience.

For scientists, the funding signal criteria identified in this research provide an overarching frame that can inform their grant preparation activities. In particular, scientists need to consider what trade-offs they are willing to make across this criteria, judgements calls that will be usefully informed by the distinction emphasised in this research between effectively addressing and over-addressing signal criteria. We hope the implications presented in this research are considered carefully. If they are not, the overall value creating potential of publicly funded science programs may diminish. This is because scientists that are repeatedly unsuccessful may choose against leading or even participating in subsequent grant funding rounds. Alternatively, they may adopt (and therefore further reinforce) some of the undesirable competitive tactics uncovered in this research, such as utilising the false investigator approach or deliberately over-addressing the signalled funding criteria. Any of these understandable reactions are sure to hinder human capital/career development among scientists and to lower the overall quality of submitted grant proposals, which will in turn diminish the value creation possibilities from publicly funded grant programmes. There is an important connection between the reputation and status of publicly funded science programmes held at the macro-level and the incentives of scientists to submit quality research proposals at the micro-level. Our research sounds a warning that this relationship needs to be managed and should not be taken for granted.

Not without limitations, our study opens up some interesting avenues for future research. We report on an in-depth case study of how funded scientists have interpreted and addressed funding criteria. Naturally, given the exploratory nature of our research, we encourage scholars to expand this research topic to other geographic locations and discipline areas beyond NZ and health-based research. We also acknowledge that we concentrated on successfully funded scientists, however, to more robustly understand potential value loss and value creation frictions arising from how signal criteria is addressed; it would be important that future studies include the views of scientists who have been unsuccessful with grant applications. There is also scope to examine in greater detail the resourcefulness contribution provided in this research. Specifically, our data did not allow us to unpack scientists’ specific resourcefulness behaviour when applying for grant funding. Future researchers should investigate different types of resourcefulness behaviour as this would help to advance understanding on the value creation goals and outcomes of grant-seeking scientists. Furthermore, future studies adopting a resourcefulness lens should incorporate the views of policymakers and funding body management at the organisational level. While we framed resourcefulness behaviour to be present among both scientists and funding bodies, our data only provided empirical evidence from the perspective of scientists. Finally, our research highlights career and in particular human capital development as areas of potential friction that can deter the overall value created through publicly funded grant programmes. Future researchers should follow the lead of Foncubierta-Rodríguez et al. (2022) and examine more closely the human capital signalled through grant applications to see what new insights can be drawn on the connection between human capital profiles and funding.

## Footnotes

1 The present research does not include HRC Explorer Grants (up to NZD$150,000) which are specifically focused on making a revolutionary change, advancing “ideas, methodologies, tools or technologies considered to be transformative, innovative, exploratory or unconventional, and have potential for major impact”. Also, Explorer Grants incorporate a lottery scheme in the allocation of funding (for more details see Liu et al., 2020).
